# Accuracy of Guided Implant Surgery in the Edentulous Jaw Using Desktop 3D-Printed Mucosal Supported Guides

**DOI:** 10.3390/jcm10030391

**Published:** 2021-01-20

**Authors:** Rani D’haese, Tom Vrombaut, Geert Hommez, Hugo De Bruyn, Stefan Vandeweghe

**Affiliations:** 1 Oral Health Sciences, Faculty of Medicine and Health Sciences, Ghent University, 9000 Ghent, Belgium; Rani.DHaese@ugent.be (R.D.); Tom.Vrombaut@ugent.be (T.V.); Geert.Hommez@ugent.be (G.H.); Hugo.deBruyn@radboudumc.nl (H.D.B.); 2 Dental Faculty, Radboud University Medical Hospital, 6525 GA Nijmegen, The Netherlands

**Keywords:** accuracy, dental implants, edentulous mandibula, guided surgery, 3D-printed surgical guide, intra oral scan

## Abstract

Purpose: The aim of this in vitro study is to evaluate the accuracy of implant position using mucosal supported surgical guides, produced by a desktop 3D printer. Methods: Ninety implants (Bone Level Roxolid, 4.1 mm × 10 mm, Straumann, Villerat, Switzerland) were placed in fifteen mandibular casts (Bonemodels, Castellón de la Plana, Spain). A mucosa-supported guide was designed and printed for each of the fifteen casts. After placement of the implants, the location was assessed by scanning the cast and scan bodies with an intra-oral scanner (Primescan^®^, Dentsply Sirona, York, PA, USA). Two comparisons were performed: one with the mucosa as a reference, and one where only the implants were aligned. Angular, coronal and apical deviations were measured. Results: The mean implant angular deviation for tissue and implant alignment were 3.25° (SD 1.69°) and 2.39° (SD 1.42°) respectively, the coronal deviation 0.82 mm (SD 0.43 mm) and 0.45 mm (SD 0.31 mm) and the apical deviation 0.99 mm (SD 0.45 mm) and 0.71 mm (SD 0.43 mm). All three variables were significantly different between the tissue and implant alignment (*p* < 0.001). Conclusion: Based on the results of this study, we conclude that guided implant surgery using desktop 3D printed mucosa-supported guides has a clinically acceptable level of accuracy. The resilience of the mucosa has a negative effect on the guide stability and increases the deviation in implant position.

## 1. Introduction

The use of a guided surgery for implant placement has been extensively investigated in the past decades, resulting in several systematic reviews [1,2,3,4,5]. Guided implant surgery is the first step in a full digital workflow for implant rehabilitation, and simplifies flapless surgery, which reduces treatment time, causes less postoperative discomfort for the patient and ensures a more predictable implant placement [6].

Guided surgery has many benefits, such as less cooling, fewer surgical complications and avoidance of poor implant positioning, which can compromise the primary stability and success of immediate loading techniques and lead to guide misfit [7,8,9]. An ill-fitting guide can be the result of errors in several steps of the process: misplacement of the prosthesis during the CBCT (Cone Beam Computed Tomography), segmentation errors in the planning software, type of fabrication technique (milled or additive) and mispositioning of the guide during surgery [10].

Differences between the virtual and actual implant position are unavoidable and will result in deviations of the implant angulation and displacement of the coronal and apical aspect of the implant [5,11]. Usually, the deviations are smallest at the coronal aspect, close to the surgical guide, where guidance is best. Angular and coronal deviation increase with increasing distance from the point of entry [5]. According to a systematic review, mucosa-supported guides resulted in maximal mean deviations of 2.19 mm at the apical aspect, 1.68 mm at the coronal aspect and 4.67 degrees in angulation [1]. The accuracy was influenced by the bone density, mucosal thickness, surgical technique, smoking, type of jaw and implant length.

Since the surgeon has little visual or tactical control during the surgery, nor the possibility to alter the location or direction of the osteotomy site, it is crucial that the surgical guide is accurate. Several factors may influence the precision of the treatment and the final implant position. The surgical guide can be teeth, bone or mucosa-supported, depending on the indication. Teeth-supported guides provide the highest accuracy, since they rely on a fixed and stable support [12]. Mucosa-supported guides depend on the thickness and resilience of the mucosa, while bone-supported guides are designed based on the CBCT data, which has an inferior resolution as compared to a gypsum cast or an intra-oral scan [13]. Improvements can be made by enlarging the support surface of the guide to reduce the degrees of freedom, or by using anchor pins to stabilize the guide [14].

Further factors include failure to follow the protocol and a cumulative error of hardware and software [5]. The latter is determined by the accuracy of the CBCT and image segmenting, the planning software and the 3D printer. In the early days, computer-guided implant planning used to be a closed workflow, where planning software and guide production were supported and controlled by the same company. Today, several companies offer planning software without guide production, which leaves the clinician the choice of whether to outsource the guide fabrication or to print the guide in-office. Gjelvold et al. [15] compared the accuracy of surgical guides from two different desktop printers and found smaller deviation at the entry point and vertical implant position for the DLP (digital light processing) printer as compared to the stereolithography printer. Chen et al. [16] compared the accuracy and dimensional stability of surgical guides produced by a lab-based polyjet printer or desktop SLA (stereolithography apparatus) 3D printers. Although the differences were clinically insignificant, the polyjet printed guides were more precise compared to the ones produced by the more economical desktop 3D printers.

The aim of this study is to evaluate the accuracy in implant positioning using mucosal supported surgical guides, produced by a desktop 3D printer. The null hypothesis is that the resilience of the mucosa has no negative effect on the accuracy of implant placement.

## 2. Experimental Section

### 2.1. Cast

**Figure 1:**
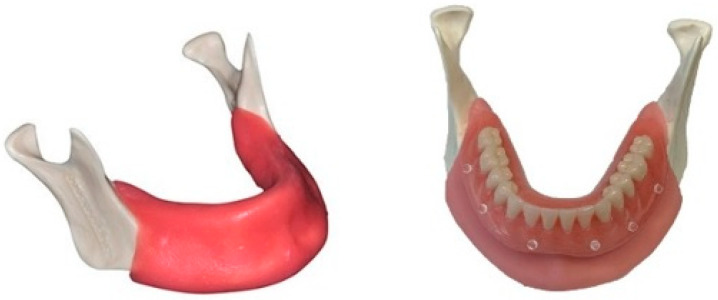
Cast with the scan-prosthesis.

Fifteen identical mandibular edentulous casts were 3D printed (Bone Models, Castellón de la Plana, Spain). The casts contained cortical and cancellous bone, classified as a Cawood and Howell Class III. A 3 mm resilient soft tissue layer, consisting of a mixture of silicones, was put on the printed cast. (Figure 1). A conventional complete denture was made on one of the casts by the dental lab and six radio-opaque markers were attached. Next, a dual-scan protocol was performed using CBCT (Planmeca 3D Pro Max, Helsinki, Finland) of the cast with the denture (54–90 kV, 1–12 mA, 400 mm isotropic voxel), and then solely of the denture at a lower kV (3.2). Despite the fact that the printed casts were identical, it was decided to scan all of them in case there were small deviations in size or shape.

### 2.2. Implant Planning

The DICOM files of the cast and denture were imported into the planning software (Exoplan^®^, Exocad GmbH, Darmstadt, Germany) and 6 dummy implants (Bone Level Roxolid, 4.1 mm × 10 mm, Straumann, Villerat, Switzerland) were planned at positions 46, 44, 42, 32, 34 and 36. Additionally, 4 anchor pins (template fixation pin 1.3 mm Ti, Straumann) were planned to provide fixation and stability for the guide. Finally, the mucosa-supported surgical guide was virtually designed and exported as an STL file. This process was repeated for each of the 15 casts.

### 2.3. Guide Fabrication

All fifteen guides were produced using a digital light processing (DLP) printer (D40, Rapid Shape GmbH, Heimsheim, Germany) and a transparent printing liquid (Nextdent SG, 3D systems, Vertex-Dental B.V., Soesterberg, The Netherlands). After printing, the guides underwent ultrasonic cleaning for 10 min in 90% ethanol. The guide sleeves were positioned in the guides prior to final curing in a UV light box for 30 min (LC-3D Printbox, 3D Systems, Rock Hill, SC, USA).

### 2.4. Implant Placement

**Figure 2:**
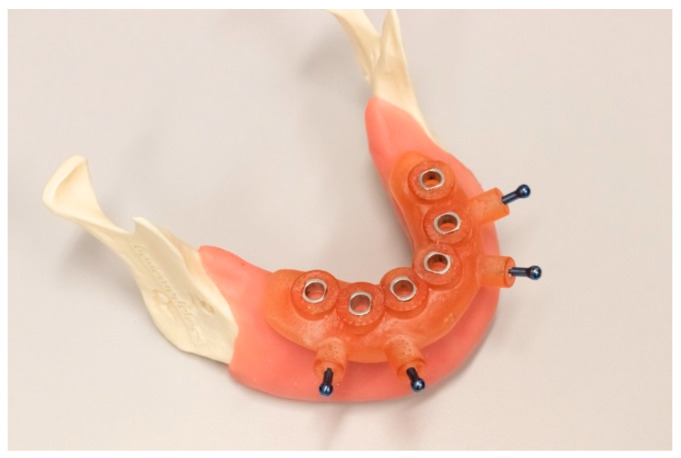
The printed guide fixed on the cast using four guide pins.

The surgical guide was positioned on the mucosa and a 1.2 mm drill was used to prepare the holes for the fixation pins (Figure 2). The surgical protocol was performed as described by the implant company. The implants were placed through the surgical guide to ensure correct vertical position of the implant.

### 2.5. Scan

After implant placement, intra-oral scan bodies were attached to the implants (ELOS accurate, Elos Medtech, Göteborg, Sweden). A digital impression was made (Primescan^®^, Dentsply Sirona, York, PA, USA) and exported as an open-format STL file.

### 2.6. Comparison

#### 2.6.1. Creating the Reference Model

Each surgical plan was imported into CAD software (Exocad Plovdiv 2.4, Exocad, Darmstadt, Germany) to create two reference models.

The reference model for the implant alignment was constructed by saving the six implant dummies as an STL file. The reference model for the mucosa alignment was constructed by saving the six implant dummies and the soft tissue layer as one STL file. The CAD file of the scan body with the corresponding implant dummy was also exported as an STL file.

#### 2.6.2. Creating the Test Model

The CAD file of the scan body and implant dummy was aligned with every scan body within the digital impressions in order to determine the implant locations (Geomagic Qualify, Geomagic Research Triangle Park, NC, USA). The first test model was created by exporting only the implant dummies as an STL file. The second test model included the implant dummies and the mucosa, and was also saved as an STL file.

#### 2.6.3. Implant and Mucosa Alignment

The corresponding reference and test model for the implant alignment were superimposed based on a “best-fit” algorithm (Geomagic Qualify, Geomagic Research Triangle Park, NC, USA). This represented the accuracy and tolerance of the guide system and sleeves, irrespective of the positioning of the guide on the mucosa. For the mucosa alignment, a “best fit” algorithm was applied only on the soft tissue layer. This outcome represents the accuracy of the guide system, including the tolerance of the drill sleeves and the position of the guide.

**Figure 3:**
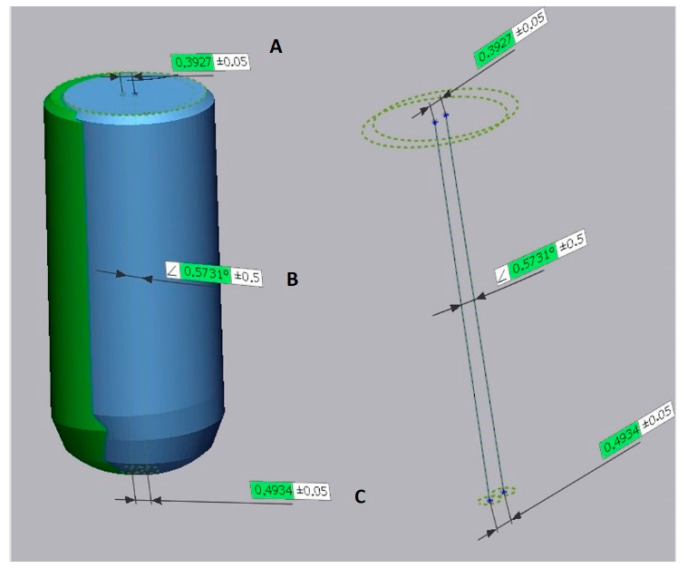
Representation of the outcome variables. A: Coronal deviation = distance between the centre of the neck of the planned implant and the centre of neck of the placed implant; B: Angular deviation = angle between the axis of the planned implant and the placed implant; C: Apical deviation = distance between the centre of the apex of the planned implant and the centre of apex of the placed implant.

**Figure 4:**
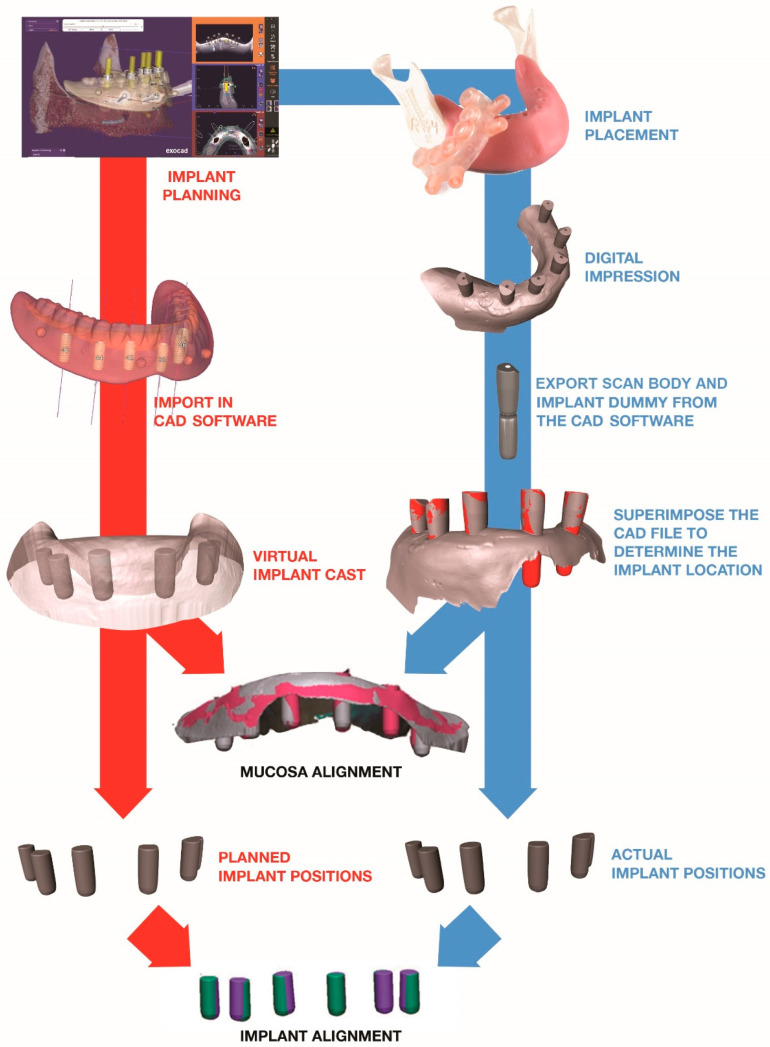
Superimposition of the planned and actual implants, using a best-fit alignment of the implants or of the mucosa.

After alignment, the angular, coronal and apical deviation of the implants were measured between the virtual plan (= reference model) and actual implant position (= test model) (Figure 3). An overview of the workflow is depicted in Figure 4.

### 2.7. Statistical Analyses

Descriptive statistics were used to evaluate the differences between planned and placed implants for both methods. Mean, standard deviation, median, min-max and 95% CI are shown.

Normality was checked with QQ plots, histograms, boxplots and the Shapiro–Wilk test. Only the coronal measurements were filed as not normally distributed in the group of the mucosa alignment. The Shapiro–Wilk test was significant for all implant alignment measurements, but after the checking of histogram, QQ plot and boxplot, it was decided to only exclude the coronal measurements as not normally distributed. The paired *t*-test was used for the normally distributed measurements, and the Wilcoxon signed-rank test was used for the coronal measurements. One-way ANOVA was used to determine the statistical differences between the individual implant locations.

Statistical analyses were done using SPSS 27, with the level of significance set at *p* < 0.05.

## 3. Results

**Figure 5:**
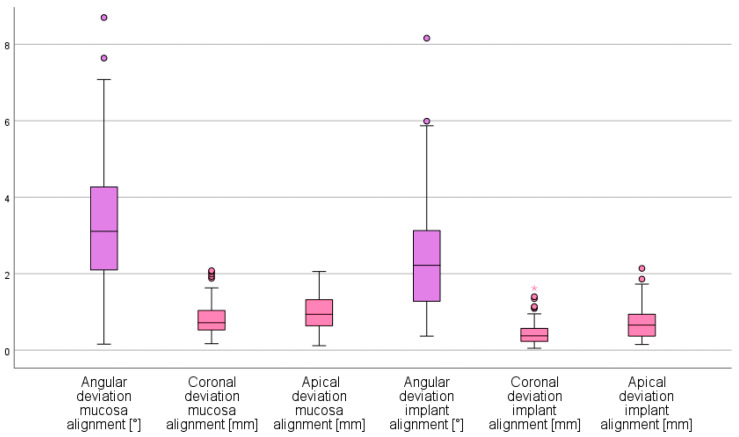
Boxplot representing the angular, coronal and apical implant deviation, based on the alignment of the soft tissue or implants. (outliers: ◯: between 1.5 and 3 × IQ (interquartile range); * more than 3 × IQ.).

In total, 90 implants were placed in 15 casts, with the use of a 3D-printed surgical guide. Angular, coronal and apical deviations are represented in Figure 5.

Mean angular implant deviation for the mucosa alignment was 3.25° (SD 1.69°; range 0.16–8.70°) the coronal deviation 0.82 mm (SD 0.43 mm; range 0.17–2.08 mm) and the apical deviation 0.99 mm (SD 0.45 mm; range 0.12–2.06 mm).

Mean angular implant deviation for implant alignment was 2.39° (SD 1.42°; range 0.37–8.16°), the coronal deviation 0.45 mm (SD 0.31 mm; range 0.05–1.62 mm) and the apical deviation 0.71 mm (SD 0.43 mm; range 0.15–2.14 mm).

All three variables were statistically significantly different between the mucosa and implant alignment (*p* < 0.001).

There were no statistically significant differences in terms of angulation (*p* = 0.683), coronal deviation (*p* = 0.782) or apical deviation (*p* = 0.189) between the different locations of the implants when considering the implant alignment. When the scans were aligned using the mucosa as a reference, no statistically significant differences were found in terms of angulation (*p* = 0.083), coronal deviation (*p* = 0.782) or apical deviation (*p* = 0.189).

The safety zone of 2 mm was exceeded apically by one implant in the implant alignment group and by three implants in the mucosa alignment group.

## 4. Discussion

The aim of this study was to evaluate the accuracy of guided surgery in the edentulous jaw, with regards to the positioning of the guide on the mucosa. This study used a new software package, which allows implant planning and guide design, while printing the guide chairside using a desktop 3D printer. In this study, a post-op digital impression was made to evaluate the accuracy instead of using CBCT.

The literature shows that the accuracy of guided implant placement in the edentulous jaw is clinically acceptable, with angular deviations in implant position between 2.4° and 4.9°, coronal deviations between 0.5 mm and 1.4 mm and apical deviations between 0.76 mm and 1.6 mm [8,12,14,17,18,19,20,21,22,23]. In our study, the respective tissue and implant angular measurements were 3.25° and 2.39°, the coronal deviations 0.82 mm and 0.45 mm and the apical deviations 0.71 mm and 0.99 mm, which correspond with these findings.

Tan et al. [24] defined the 2 mm safety zone as 1 mm bone volume required for the buccal blood supply and 1.1 mm for the error related to the surgical technique. This was exceeded four times in our study, with a maximal apical deviation of 2.14 mm [2,8,9]. Three out of the four deviations over 2 mm were in the tissue alignment group. To avoid complications, clinicians should always respect a 2 mm distance to critical structures. The four measurements that exceeded the 2 mm zone were on positions 32, 34 and twice on position 42. Although the largest deviations were thus found in the anterior region, no significant differences in deviation were found between the implant locations for both alignments.

The jaw and its anatomical shape might affect the accuracy of guided implant surgery. In a cadaver-based study, Petterson et al. [23] found that the maxillary treatment was significantly more accurate than the mandibulary. This finding was confirmed in a clinical study by Cassetta et al. [25]. Both studies related this to the larger supporting surface of the maxilla, which increased guide stability. Accuracy was also improved when the guide was fixated with fixation pins, which were also used in our study [25]. With respect to the mandible, Lin et al. [19] reported comparable results in their in vitro study, with a mean angular deviation of 2.70°, mean coronal deviation of 0.5 mm and a mean apical deviation of 0.99 mm. Despite the found difference in accuracy between mandibula and maxilla, it is yet to be determined if it has any clinical significance.

Deeb et al. [26] investigated the use of a desktop 3D printer versus a laboratory-made surgical guide and found no differences in terms of accuracy of implant placement. In-office printing has the advantage of being more economical and less time-consuming, and eliminates the need of communicating with the laboratory. On the other hand, the clinician has to learn to work with the software, and needs to invest in the hardware, which also requires maintenance.

Most studies which evaluate the accuracy of guided implant surgery use a second post-operative CBCT to compare the actual implant position with the virtual planned implant [9,27,28]. This holds some disadvantages, such as the additional radiation for the patient and the presence of beam hardening, which is caused by the implant and disturbs the outline of the implant [29]. Since the resolution of CBCT is dependent on its voxel size (200 µm), the level of detail is limited [30,31]. Son et al. [32] introduced a new technique to evaluate the accuracy of guided implant placement by comparing a post-operative intra-oral scan of the implant with the virtual planned implant. This approach eliminates the radiation, beam hardening and errors from patient movement during post-operative CBCT scanning. Skjerven et al. [33] compared both techniques and concluded that both yielded comparable results. Brandt et al. [34] also compared both evaluation methods and found that measurements on a 3D surface mesh were more accurate compared to the measurements on a CBCT.

To our knowledge, this is the first study that evaluated the accuracy of guided implant surgery in an edentulous arch using optical scanning instead of a post-op CBCT. Some studies [9,24,35] have used this approach in partially edentulous arches, using tooth-supported guides. Angular, coronal and apical deviations respectively varied between 1.6° and 4.8°, 0.42 mm and 1.2 mm and 0.32 mm and 1.8 mm.

In the current study, outcome measurements were based on two different alignments. The first alignment was merely based on the mutual position of the implants, without taking the location in the jaw into consideration. This bypasses the positioning error of the surgical guide and only provides information regarding the position of the implants in relation to the guide. The second alignment was based on the outline of the mucosa and represents the spatial position of the implants in relation to the jaw and includes the positioning error of the guide. In this case, displacement of the guide will not affect the mutual position of the implants, but will result in a different position of the implants within the jaw. Larger deviations were found when the alignment was done with the mucosa as a reference, which demonstrates that a significant error may be induced when positioning the guide. This means that the null hypothesis can be rejected and confirms the findings by D’haese et al. [14], who also investigated the influence of guide position and came to similar conclusions.

Finding the correct position of the surgical guide can be challenging due to the flexibility and resilience of the mucosa. The pressure exerted during the surgery in combination with the resilient mucosa will cause the surgical guide to move or rotate [17]. Especially in the edentulous arch, the guide displays more rotational freedom compared to partially edentulous patients [14]. Ochi et al. [36] demonstrated that the pressure on the mucosa will be different when the patient bites firmly during the CBCT scanning or when the surgeon positions and fixes the guide. Thus, the final position of the guide might be slightly different compared to the position of the scan-prosthesis. Although our study was performed in vitro, the 3D-printed cast had a flexible mucosal layer which mimicked the natural resilience and thus compromised the correct positioning of the guide.

The thickness of the mucosa plays a significant role in this, since a thicker mucosa leads to more deviation of the position of the surgical guide [25]. In this study, the mucosa had an overall equal thickness of 3 mm. This is rather thick when compared to in vivo measurements of the mucosal thickness in the mandibula, which could have led to an overestimation of the deviations [37]. A thicker mucosa is common in smokers and is known to negatively influence the guide positioning and accuracy of implant placement [25,38].

The fit and correct position of the surgical guide also depends on the image processing. This means that the choice of grey density threshold determines the form of the tissue [17]. When this is chosen incorrectly by the operator, it will result in an incorrect virtual cast and deformation of the guide. This will decrease its fit and result in incorrect positioning of the guide and implants. Additionally, the manual placement of the sleeves in the guide might be subjected to errors [39].

Despite the promising results in this study, there are still some limitations related to the nature of this research. Marliere et al. [6] reported that casts guarantee a greater stability of the surgical guides and more accurate implant placement when compared to an in vivo situation.

In the present study, there is no interference of the floor of the mouth, tongue, buccal mucosa, or buccal sulcus, which may lead to greater instability of the guide. Additionally, lack of visibility caused by bleeding or limited mouth opening might negatively affect the guide positioning and accuracy of implant placement [9]. Therefore, a clinical study is still required to confirm these results.

## 5. Conclusions

Guided implant surgery using desktop 3D-printed mucosa-supported guides had a clinically acceptable level of accuracy. The resilience of the mucosa had a negative effect on the guide stability and increased the deviation in implant position.

## Data Availability

Data are available from the corresponding author upon request.
